# Interaction of sublingual glyceryl tri-nitrate administration on single muscle contraction-induced hyperemia: potential insight into the role of the skeletal muscle pump

**DOI:** 10.3389/fphys.2026.1838894

**Published:** 2026-07-08

**Authors:** Timothy R. Rotarius, Jakob D. Lauver, Barry W. Scheuermann

**Affiliations:** 1 Department of Kinesiology, Coastal Carolina University, Conway, SC, United States; 2 College of Health, Ball State University, Muncie, IN, United States; 3 School of Exercise and Rehabilitation Sciences, University of Toledo, Toledo, OH, United States

**Keywords:** blood flow, rapid vasodilation, single contraction, skeletal muscle pump, vascular conductance

## Abstract

**Purpose:**

Much debate exists about the relative contribution of the skeletal muscle pump and rapid vasodilation to exercise hyperemia. This investigation examined the contribution of the skeletal muscle pump to the immediate increase in muscle blood flow (MBF) following a single muscle contraction.

**Methods:**

Prior vasodilation using glyceryl tri-nitrate (GTN) abolished the impact of contraction-induced vasodilation following a single contraction. Eight healthy individuals completed a series of three, single muscle contractions at 10% of MVIC, each separated by 90 seconds of recovery for the control (CON) condition. For the GTN condition, participants were given a 0.4 mg dose of GTN sublingually and given enough time for brachial artery diameter to reach a new steady-state. The single contraction model from CON was repeated for GTN condition. Each condition was repeated twice for a total of 6 contractions. Brachial artery diameter and blood velocity were obtained using an ultrasound system operating in Duplex mode.

**Results:**

Brachial artery diameter was significantly greater following GTN administration prior to contraction (CON: 0.48 ± 0.07 cm; GTN: 0.56 ± 0.07 cm, p< 0.05, *d =* 1.15). Forearm blood flow (FBF) was significantly lower (greater retrograde flow in GTN) during the contractile phase following GTN administration (CON: 0.75 ± 38.23 mL/min; GTN: -100.87 ± 45.00 mL/min, p< 0.05, *d =* 2.43), and significantly greater immediately (within 1 s) following the release of contraction (CON: 160.80 ± 72.91 mL/min; GTN: 232.87 ± 102.78 mL/min, p< 0.05, *d =* 0.81).

**Conclusion:**

This suggests that the immediate increase in FBF with GTN administration is due to rapid vasodilation and mechanical contraction.

## Introduction

Following a single contraction of the forearm muscles, there is an immediate (within 1 s) increase in forearm blood flow (FBF) that reaches a peak value after approximately 10 seconds (Corcondilas et al., 1964). This increase has been attributed to the propulsion of blood from the vasculature following the muscle contraction (Laughlin, 1987) or rapid vasodilation of the microvasculature. However, the relative contribution of these mechanisms remains unclear, as previous studies have not fully isolated the mechanical effects of the skeletal muscle pump from vasodilatory influences. Sheriff and associates have found increases in muscle blood flow during exercise that are proportional to contraction frequency, consistent with a muscle pumping effect (Sheriff et al., 1993; Sheriff and Hakeman, 2001; Sheriff, 2003); while others have concluded that a rapid vasodilation occurs within the first 2 s of voluntary contraction, leading to an increased FBF (Tschakovsky et al., 1996). Attempts to isolate the predominant mechanism contributing to the increase in FBF following a single muscle contraction have utilized various experimental approaches including the blockade of various known vasodilators. For example, Brock et al. (1998) used L-NMMA and atropine to block nitric oxide (NO) production and acetylcholine receptors, respectively. More recent studies by Crecelius et al. (2013) used BaCl_2_ and ouabain to block K^+^ channels, which are also involved in regulating vasodilation. While the inhibitors used in these studies were effective in blocking the intended vasodilators as demonstrated by the subsequent attenuation of contraction-induced vasodilation, the administration of the inhibitors did not abolish the blood flow response, suggesting that some other mechanism (i.e. the skeletal muscle pump) was responsible for the increase in MBF.

Glyceryl tri-nitrate (GTN) is a known potent vasodilator due to its ability to donate NO when metabolized by aldehyde dehydrogenase II (Chen and Stamler, 2006) thereby bypassing the shear-induced increase in NO production generated by increases in blood flow. Data from our laboratory has shown that GTN administration can increase resting brachial artery diameter by up to 33% (Stacy et al., 2013). Because the increase in metabolite production due to muscle contraction is thought to cause a significant and rapid vasodilation, by administering GTN and pre-vasodilating (or priming) the artery, any increase in muscle blood flow following a single muscle contraction may be attributed to the skeletal muscle pump and not changes in arterial diameter. To our knowledge, only a few studies have infused a potent vasodilator prior to the onset of muscle contractions (Hamann et al., 2003; Rotarius et al., 2021; Shepherd et al., 2016). However, not only was one study performed in the dog hindlimb (Hamann et al., 2003), but all of these studies utilized multiple contractions. Previous research has shown that blood flow during dynamic contractions is not a steady state, but rather oscillatory with peak flows reached during the relaxation of the exercising muscle (Saltin et al., 1998; Tschakovsky et al., 2004; Walløe and Wesche, 1988); thus, the dynamic contraction model would preclude the examination of this oscillatory pattern and introduces certain reflex effects that could influence blood pressure (Hamann et al., 2004). Therefore, the single contraction model is a paradigm that allows for the examination of the oscillatory pattern of blood flow without the confounding effects of humoral factors.

To our knowledge, this is one of the first studies to examine the effect of feed artery priming (i.e. prior vasodilation) on FBF following a single forearm contraction. By vasodilating the brachial artery prior to muscle contraction, it is thought that the mechanical effects imposed by muscle contraction (i.e. the muscle pump) would be isolated. Unlike previous studies that infused vasodilators during dynamic exercise or in animal models, the present investigation focuses on a single voluntary contraction in humans, attempting to eliminate the influence of confounding effects of oscillatory flow and exercise metaboreflexes. It was hypothesized that following a single muscle contraction, blood velocity post-contraction would be decreased consequent to the increase in arterial diameter, so that FBF between the two conditions would not be different. It was speculated that the effectiveness of the skeletal muscle pump would be diminished due to a greater resting blood flow, indicating a greater reliance of vasodilation in the GTN condition.

## Methods

### Subjects

Eight (8), young (age range 18–40 years old), healthy individuals volunteered to participate in this study. Power calculations were performed using the G*Power software. The most conservative effect size (d = 0.6) indicated that a sample size of 8 subjects would be sufficient when α = 0.05 and β = 0.80. The sample size recruited in this study is comparable to previous research conducted in our laboratory (Rotarius et al., 2021). Subjects were excluded from this study if they had any known cardiovascular or metabolic diseases or, if they were currently prescribed sildenafil citrate, tadalafil, or other medications that lower blood pressure. In addition, any individuals that experience frequent migraines (1–2 times per month) were excluded from participation in the study. Subjects were considered recreationally active based on responses to a physical activity questionnaire. Subjects were instructed to refrain from any moderate to vigorous physical activity while enrolled in the study, and to avoid consuming caffeinated beverages on scheduled study days. Each subject was informed of all testing procedures as well as the risks and discomforts associated with participating in the study and were given an opportunity to ask questions prior to providing written informed consent. This study was approved by the Human Subjects Committee of the Institutional Review Board at the University of Toledo and was conducted in accordance with the Declaration of Helsinki.

### Familiarization session

Prior to data collection, each subject participated in a familiarization-preliminary data collection session that included anthropometric measurements and subject screenings for clearly identifiable brachial artery borders. Subjects were asked to perform three maximal contractions of the forearm (MVC) to determine the weight used for the single muscle contraction. Subjects gripped a standard handgrip dynamometer (TKK5401 Grip-D, Takei Scientific Instruments Co., Niigata, Japan) and maximally contracted for 5 seconds, after which the value on the digital display was recorded. Subjects were given 2 minutes of rest between each MVC. The average of three trials was calculated as each subject’s MVC. Subjects were then asked to lay down in the supine position with their arm outstretched at a 90° angle for brachial artery screening. This was done to both familiarize the subject to testing procedures and to ensure that a satisfactory image of the brachial artery could be obtained and analyzed.

Lastly, subjects underwent a glyceryl tri-nitrate (GTN) administration procedure. Due to the short half-life of GTN, all subjects were given a 0.4 mg dose of GTN sublingually and brachial artery diameters were measured continuously until the increase in vessel diameter reached a new steady-state. Brachial artery diameter and the time to peak vasodilation were recorded and used for subsequent testing.

### Experimental protocol

Following the familiarization session, subjects were asked to return to the Cardiopulmonary and Metabolism Research Laboratory on 4 separate days for handgrip exercise and GTN administration protocol. Subjects were asked to lie down in the supine position for 20 minutes with their dominant arm outstretched to a 90° angle and gripping a custom-made handgrip dynamometer. All testing was performed in a quiet, temperature-controlled room (22-24°C) with the lighting reduced for optimal visualization of the vessel image and blood velocity profile.

Each single contraction trial consisted of an initial baseline image (including the longitudinal view of the vessel and the blood velocity profile) that was recorded for 30 s followed by a single contraction of the forearm at 10% of the subjects’ MVC. The single contraction was performed using a custom-built handgrip dynamometer, with the load attached to the end of a pulley system designed to move 75 mm. Ninety seconds of recovery were recorded following the single contraction. This procedure was repeated two more times for a total of three contractions.

For the GTN condition, an initial baseline image was recorded for 30 s, at which point subjects were administered GTN and artery diameter and blood velocity were measured continuously until artery diameter reached the new steady-state obtained from the preliminary trial day. After the new diameter steady-state was achieved, subjects performed three, single contractions with 90 s of recovery between each contraction. Both the CON and GTN conditions were repeated a second time, on a separate day, for a total of six contractions for each condition.

### Brachial artery diameter analysis

Both resting HR and BP were measured using an automated finger plethysmography system (Finometer Model 1, Finapres Medical Systems, The Netherlands) that continuously measured each variable throughout the duration of the protocol. Total peripheral resistance (TPR) was calculated using Equation 1:

(1) $$\text{TPR }=\text{ MAP}/\dot{\text{Q}}$$

where MAP is equal to the mean arterial pressure, and Q̇ is equal to the cardiac output.

Longitudinal images of the brachial artery were obtained using B-mode two-dimensional ultrasonography with a multiple-frequency linear array probe (Model L8-2; center frequency of 7.5 MHz) attached to an ultrasound system operating in Duplex mode (z.one ultra, Zonare Medical Systems Inc., Mountain View, CA). The ultrasound probe was placed approximately 7–10 cm proximal to the antecubital fossa on the medial aspect of the upper arm and was secured in place using a custom-built articulating arm and clamp once the optimal image was found. Recording of the images for measuring vessel diameter and the Doppler ultrasound measurements of blood velocity were achieved using the same transducer operating simultaneously in 2D echo and pulse wave Doppler modes. All blood velocity measurements were determined with the transducer positioned to maintain an angle of insonation of ≤ 60°for the artery of interest. The gate was adjusted to the width of the artery to ensure that the sample volume included the near and far wall borders and remained centered in the lumen of the vessel.

The image of the brachial artery and the corresponding blood velocity profiles were recorded at a sampling frequency of 30 Hz using a digital imaging frame grabber (DVI2USB 3.0, Epiphan Systems Inc., Palo Alto, CA) and acquisition software (Cardiovascular Suite 2.1, Quipu srl, Pisa, Italy). The video files (vessel wall and blood velocity recordings) were stored on a computer for offline analysis at a later time. Forearm blood flow (FBF) was calculated using Equation 2:

(2) $$\text{FBF }\left(\text{mL}/\text{min}\right)\;=\text{ MBV }*\text{ π}{\text{r}}^{2}*\;60\text{ s}$$

where FBF is mean forearm blood flow, and MBV, mean blood velocity, equals the difference between antegrade and retrograde blood velocities.

Forearm vascular conductance (FVC) was calculated using Equation 3:

(3) FVC (mL/min/mmHg) = FBF/MAP * 100

For each single contraction trial, arterial diameter and blood velocity (and shear rate) were determined using an investigator-independent process including on-screen calibration of diameter and blood velocity images, identification of the vascular wall and blood velocity regions of interests (ROI) and the automated detection of vascular borders and blood velocity envelop profile. This interactive process, requiring the operator to identify the ROI for the vessel diameter and the blood velocity waveform profile, is designed to ensure that the quality of the vessel image and the blood velocity waveform is optimized allowing for the accurate assessment of the GTN or exercise response (Gemignani et al., 2007). Once the ROI for the vessel diameter and the blood velocity waveform was established at the onset of each trial, the same ROI was applied to every frame in the trial (i.e. the arterial diameter and blood velocity were determined for the same vessel segment and sampling region). The software utilizes the information within the ROI to “learn” the identification of the vessel borders using an optimized graphical search algorithm detection approach, which is based on the varying pixel intensity defining the image on the computer monitor within the ROI. As expected, vascular borders may vary appreciably at rest and during muscle contractions due to systolic and diastolic changes in blood pressure and thus, parameters from the initial sample within the ROI are sequentially applied to subsequent frames during the analysis. During the analysis, a quality control mechanism is utilized by the iterative algorithm that uses a gradient and shape tolerance parameter that minimizes the vessel border segment variability while maximizing the vessel border segment for reliability and robustness. These parameters indicate an acceptable difference between the wall borders of the vessel (i.e. gradient tolerance) and the allowed quality of the variability of each of the borders forming the vessel wall (i.e. shape tolerance). The overall diameter for each frame is calculated by taking the computed distance of two points on the borders defining the near and far arterial wall using a set of diameters (where diameter = d1, d2,…dn) within the defined ROI. The actual number of diameter samples included in the determination of the diameter reported for each specific frame is dependent upon the width of the ROI that is employed for each single contraction trial. The sequential measurement of diameters used to determine the overall diameter for that frame along with the standard deviations of those serial measurements yield a confidence interval for each frame providing the operator with an indication of the quality of the image analysis.

### Blood velocity analysis

Beat-by-beat blood velocity analyses were carried out using FloWave.US (*see* Coolbaugh et al., 2016 for full description). FloWave.US is hosted in a version control repository and can be executed in MATLAB (The Mathworks, Natick, MA). In brief, FloWave.US can analyze digital video recordings of duplex or B-mode ultrasound screen captures. From these videos, FloWave.US can estimate time-averaged mean (TAMean) velocities from the Doppler pulse-wave spectrum. First, video images are cropped to a ROI around the Doppler pulse wave spectrum. Within this ROI, a pixel’s color intensity is calculated if it is within the threshold of either the TAMean color overlay or the Doppler pulse wave spectrum. Next, the location of the maximal pixel intensity from the TAMean color overlay or the Doppler envelope boundaries is identified. The pixel position data are then converted to a positive (antegrade) or negative (retrograde) velocity (cm/s) according to the pixel scaling factor and its relative position to the zero-velocity baseline. Data from each video file was then exported to a set of comma-separated variable files for later offline analysis.

The intra-cardiac cycle blood flow was then calculated using the mean BA diameter and the beat-by-beat blood velocities from MATLAB using **Equation 2**.

### Data processing

Each single contraction was ensemble-averaged so that the time point at which the greatest retrograde blood velocity occurred was considered Time = 0. The arterial waveform from finger plethysmography was fed into an analog-to-digital converter (PowerLab 8/35, ADInstruments, Sydney, Australia) and analyzed for systolic, diastolic, and mean arterial pressures using LabChart (ADIstruments, Sydney, Australia). Data from the Doppler video analysis were time-aligned with the central hemodynamic variables and were then averaged into 1-s bins for statistical comparison.

### Statistical analysis

A paired t-test was conducted to determine differences in brachial artery diameter prior to the onset of a single contraction between the CON and GTN conditions. Linear mixed-effects models were used to examine the effects of condition (CON vs. GTN), time (−10 to 20 s relative to contraction), and their interaction on forearm blood flow (FBF) and forearm vascular conductance (FVC). Condition, time, and the condition × time interaction were specified as fixed effects. Subject was included as a random effect with a random intercept to account for repeated observations within individuals. Repeated observations across time were modeled using a first-order autoregressive [AR(1)] covariance structure to account for temporal autocorrelation between adjacent time points. Models were estimated using restricted maximum likelihood (REML), and denominator degrees of freedom were computed using the Satterthwaite approximation. A two-way analysis of variance (ANOVA) with two repeated measures (Condition, Time) was conducted to determine differences in central hemodynamic variables (HR, SV, and MAP) between the CON group and the GTN group. Residuals from the ANOVA were assessed using a Shapiro-Wilks test to determine normality. Mauchly’s tests of sphericity were conducted and the Greenhouse-Geisser methods was used to correct degrees of freedom for significant main and interaction effects. A Tukey’s *post-hoc* test was used, if needed. All data is presented as the mean ± SD. Statistical significance was set *a priori* at p< 0.05. Effect sizes for pairwise comparisons were calculated using Cohen’s *d(z)* for repeated-measures designs (small effect< 0.4, medium effect = 0.40 – 0.75, large effect = 0.75 – 1.1, very large effect = 1.1 – 1.45, and huge effect > 1.45), as this approach accounts for within-subject variability and is appropriate for dependent samples (Cohen, 2013). Data was analyzed using Sigma Plot software (SigmaPlot 13.0, Systat Software, Inc., San Jose, CA).

## Results

Eight, healthy males (29.6 ± 9.4 yrs.; 177.0 ± 6.2 cm; 90.9 ± 20.7 kg) participated in this investigation.

### Blood flow response to a single contraction

**Figure 1:**
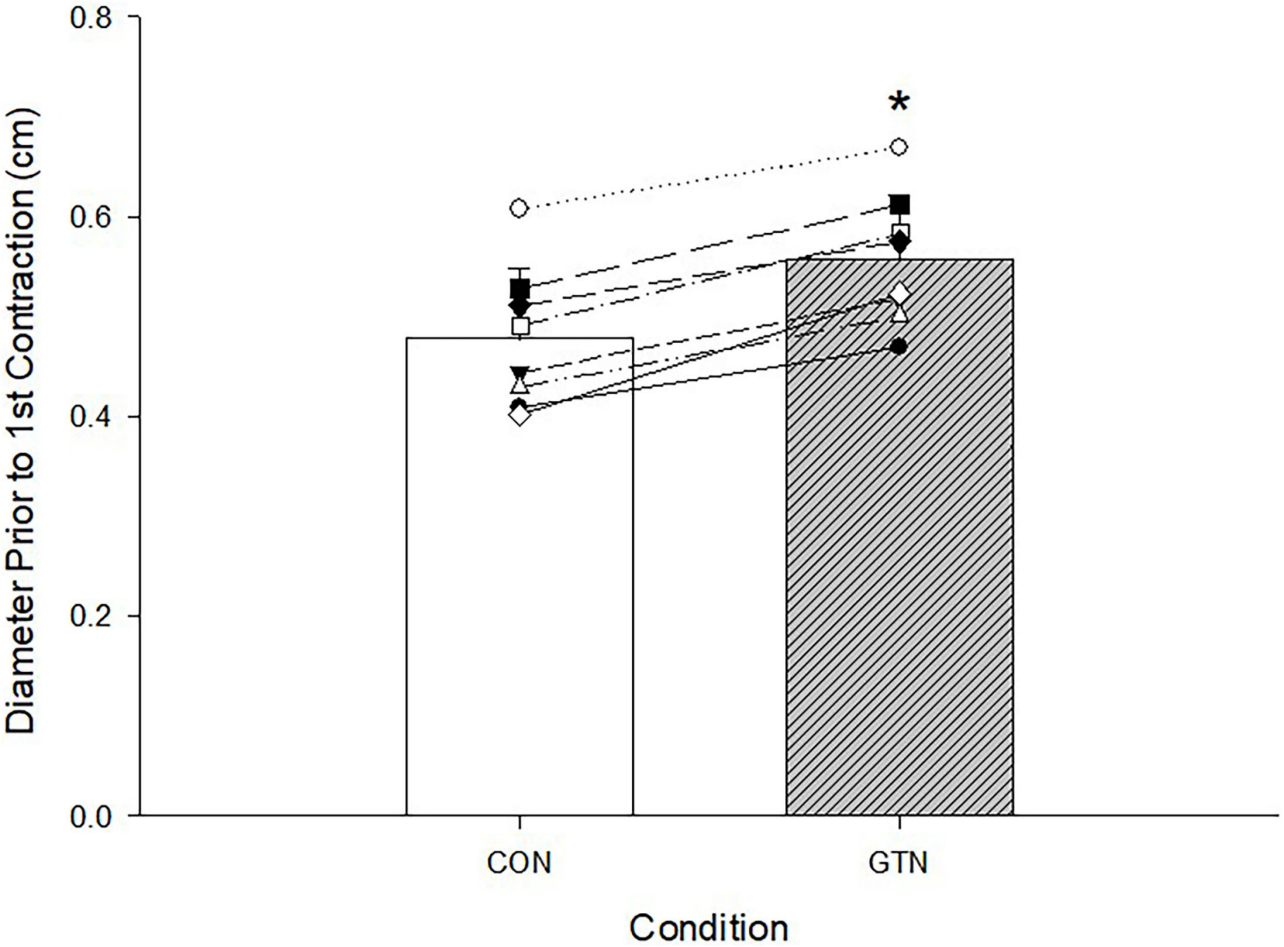
Group mean [Bar (± SD)] and individual data points for brachial artery diameter prior to single contractions. Error bars represent standard deviations. * indicates significance at p< 0.05.

The paired t-test determined that there was a significant difference in brachial artery diameter prior to the onset of a single contraction between CON (0.48 ± 0.07 cm) and GTN (0.56 ± 0.07 cm), p< 0.05; *d* = 1.15 (**Figure 1**).

**Figure 2:**
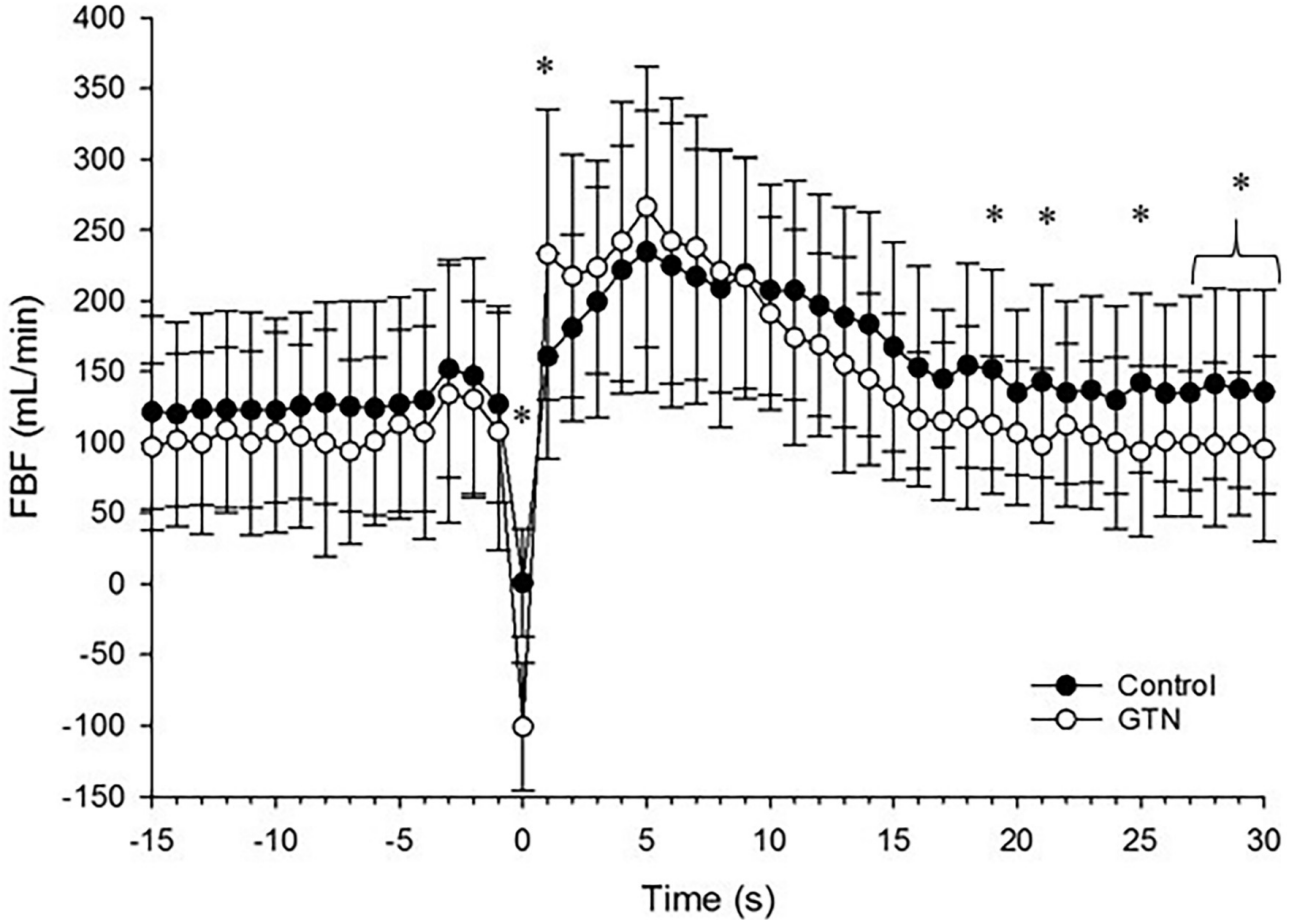
Group mean (± SD) response for forearm blood flow (FBF) following a single muscle contraction. Error bars represent standard deviations. Raw data was compiled into 1-s averages and ensemble-averaged over the 6 contractions. * indicates significance at p< 0.05.

Linear mixed-effects modeling revealed a significant main effect of time on FBF (F(30, 247.57) = 45.29, p<.001) and a significant condition × time interaction (F(30, 240.04) = 4.67, p<.001). However, there was no main effect for condition. Further *post hoc* analysis showed that FBF in GTN was significantly lower during the contraction phase (CON = 0.75 ± 38.23 mL/min; GTN = -100.87 ± 45.00 mL/min, p< 0.05; *d(z) =* -1.88) and significantly greater immediately (within 1 second) following the release of contraction (CON = 160.80 ± 72.91 mL/min; GTN = 232.87 ± 102.78 mL/min, p< 0.05; *d(z) =* 1.20) (**Figure 2**). Any significant differences in FBF following a single contraction after 1 s were mostly resolved.

### Forearm vascular conductance

**Figure 3:**
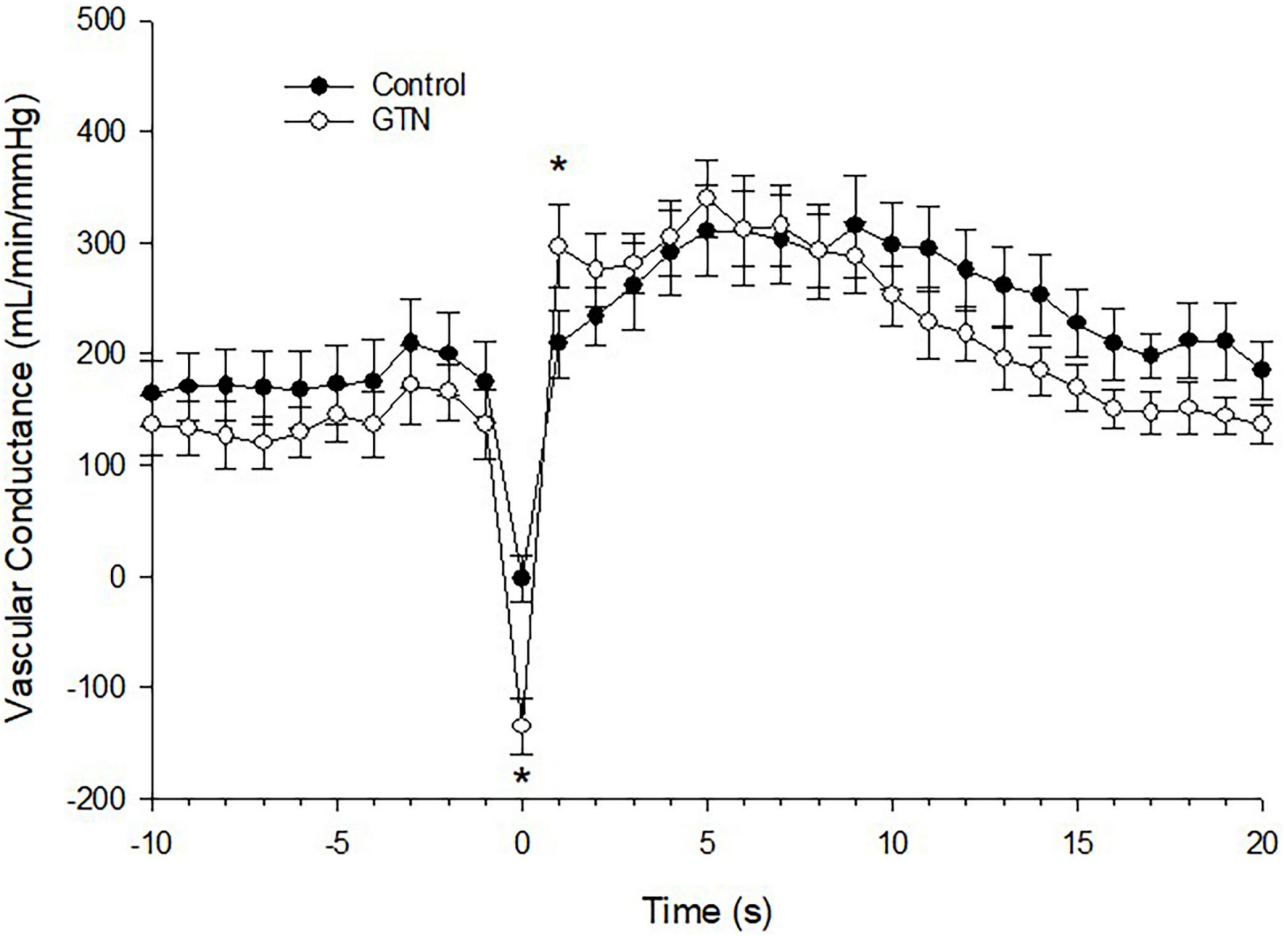
Changes in vascular conductance following a single muscle contraction. Error bars represent standard deviations. Vascular conductance was ensemble-averaged over the 6 contractions and is presented as 1-s averages. * represents significance at p< 0.05.

Linear mixed-effects modeling revealed a significant main effect of condition on FVC (F(1, 30.67) = 5.53, p = .025), a significant main effect of time (F(30, 258.13) = 43.72, p<.001), and a significant condition × time interaction (F(30, 245.11) = 4.27, p<.001). Further *post hoc* analysis showed that FVC in GTN was significantly lower during the contraction phase (CON = -2.3 ± 21.0 mL/min/mmHg; GTN = -135.6 ± 25.7 mL/min/mmHg, p< 0.05; *d(z) =* -1.55) and significantly greater immediately (within 1 second) the release of contraction (CON = 208.8 ± 30.2 mL/min/mmHg; GTN = 296.5 ± 37.4 mL/min/mmHg, p< 0.05; *d(z) =* 1.03) (**Figure 3**). Any significant differences in FVC following a single contraction after 1 s were mostly resolved, yet FVC was significantly greater in CON at 19 s, 21 s, 25 s, and 28–30 s post-contraction.

### Central hemodynamics

**Figure 4:**
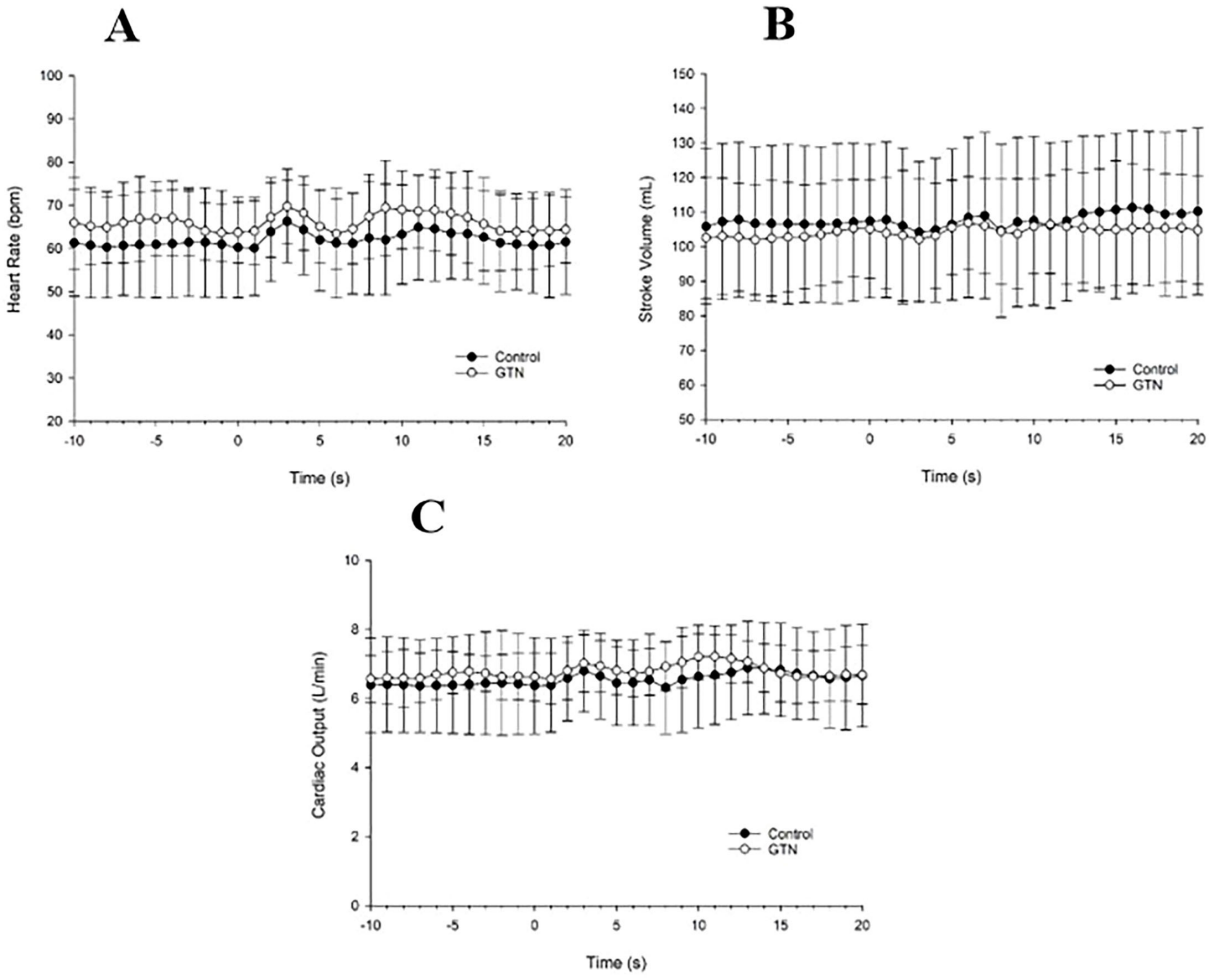
Changes in heart rate **(A)**, stroke volume **(B)**, and cardiac output **(C)** following a single muscle contraction. Error bars represent standard deviations. Central hemodynamics was ensemble-averaged over the 6 contractions and is presented as 1-s averages.

**Figure 5:**
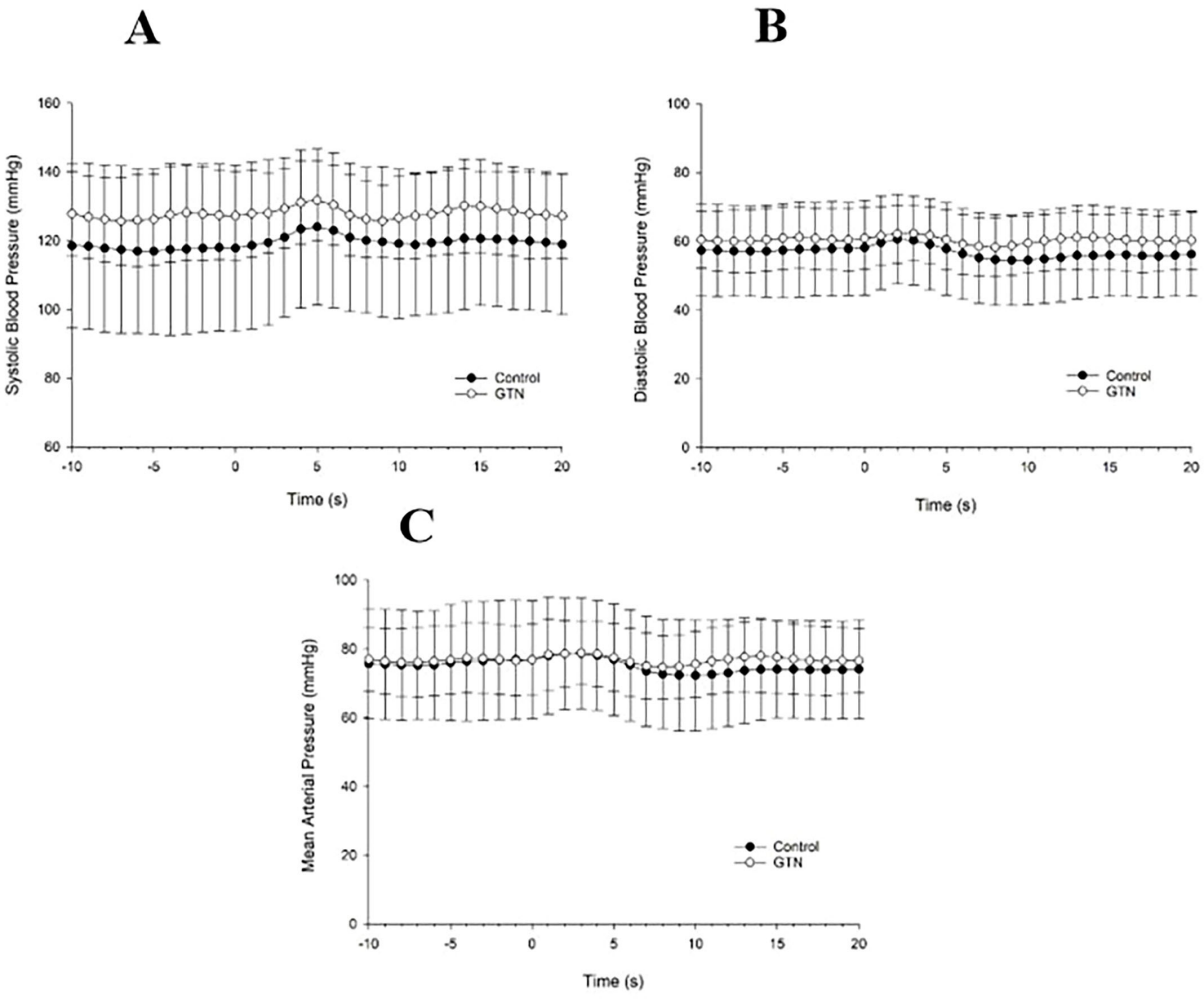
Changes in systolic **(A)**, diastolic **(B)**, and mean arterial **(C)** pressure following a single muscle contraction. Error bars represent standard deviations. Blood pressure was ensemble-averaged over the 6 contractions and is presented as 1-s averages.

There was a significant main effect for time for both HR and SV (p< 0.05). This ultimately led to a significant main effect of time for cardiac output (p< 0.05) and an interaction effect for cardiac output (p< 0.05). However, there was no main effect for condition for all three variables (**Figure 4**). Similar to HR, SV, and CO, there was a significant main effect for time for SBP, DBP, and MAP (p< 0.05). There was also a significant interaction effect of time x condition for DBP and MAP, but not for SBP. There was no main effect of condition for measures of blood pressure (**Figure 5**). Total peripheral resistance was not significantly different across all time points, and there was no significant difference in TPR between conditions (CON vs. GTN).

## Discussion

The purpose of this investigation was to determine the effect of GTN administration (pre-vasodilation) on FBF following a single forearm contraction. In doing so, we attempted to isolate any mechanical effects imposed by muscle contraction on the blood flow response to a single contraction. Contrary to our hypothesis that FBF would not differ between conditions due to the offsetting effects of increased arterial diameter and reduced blood velocity, FBF was transiently greater immediately post-contraction following GTN administration. Additionally, FBF was elevated above baseline values for both conditions, with peak FBF occurring approximately 5 s post-contraction. For the GTN condition, following muscle contraction, FBF was significantly greater than baseline values and significantly different from CON at 1 s. However, the difference in FBF between CON and GTN disappeared after 2 s post-contraction. Additionally, FVC was significantly greater immediately post-contraction following GTN administration compared to CON. These findings provide evidence that the skeletal muscle pump may still be intact under prior vasodilatory conditions, impacting FBF during contraction and resulting in a greater retrograde flow, but rapid vasodilatory mechanisms may have been augmented following GTN administration, thus resulting in augmented post-contraction hyperemia.

### Blood flow following a single contraction

Prior investigations have shown that blockade of NO, acetylcholine, Na^+^-K^+^-ATPase, and K_IR_ (potassium inward-rectifying) channels decrease FBF following a single contraction (Brock et al., 1998; Crecelius et al., 2013). This suggests that NO plays a role in modifying mechanical factors that contribute to the FBF response to a single contraction. It also suggests that these pathways play a major role in muscle blood flow regulation in humans and that a mechanically-induced vasodilation can explain most of the FBF response. The results of the present investigation seem to support the idea that these rapid vasodilatory mechanisms are predominantly responsible for the regulation of muscle blood flow immediately (within 1 s) following a single contraction. In fact, these results support that rapid vasodilatory mechanisms can be augmented with prior vasodilation and account for further augmentations in FBF from 1–5 s post-contraction. This is in partial agreement with the results of Dillon et al. (2020), which have shown that continuous, exogenous adenosine (ADO) and adenosine tri-phosphate (ATP) do not blunt the peak hyperemic and vasodilator responses to a single contraction. These authors purport those other metabolites such as nitric oxide, prostaglandins, and potassium worked synergistically with vasodilator infusion. The results of the present investigation show that despite a significant NO-mediated vasodilation prior to single contractions, FBF was approximately 45% greater in GTN immediately following contraction (within 1 s) compared to CON. Additionally, FVC was significantly greater in GTN post-contraction compared to CON. As explained in Dillon et al. (2020), FVC is a primary index of vasodilation as it is linearly related to blood flow, while also accounting for changes in MAP. This would indicate rapid onset vasodilation was augmented post-contraction following GTN administration and greatly contributed to the post-contraction hyperemia. The rationale for employing a single contraction model is that the evaluation of the blood flow response following a single contraction is unhindered by the potentially confounding effects of the baroreflex and the accumulation of metabolic by-products (Hamann et al., 2003). Thus, it can be speculated that any increases in FBF following a single contraction with GTN are attributable to the skeletal muscle pump. However, this may not have entirely been the case, as evidenced by the differences in FVC between conditions.

It is possible that GTN administration, while known for its systemic vasodilatory effects, also caused terminal arteriolar vasodilation to occur within the muscle. Marshall and Tandon (1984) have shown that terminal arterioles are capable of vasodilating within 2 s of muscle contraction. However, Wunsch et al. (2000) found that rapid vasodilation can take nearly 4 s to occur. Therefore, it is likely that any increases in blood flow within 2–4 s is likely due to rapid vasodilation and reduced peripheral resistance distal to the conduit artery (Perrin et al., 2026). Our results are in partial agreement. In the present investigation, we found a significant increase in FBF and FVC within 1 s of a single contraction after GTN administration, when vasodilation was physiologically maximal. Thus, one can speculate that the initial increase in FBF was due in large part to augmented rapid onset vasodilation, and thus, a reduced peripheral resistance. This augmentation, however, was quickly reconciled (after 2 s), suggesting a hierarchy of vascular control that is significantly different between the conduit artery and the microvasculature. Evidence for this has been shown through the use of diffuse correlation spectroscopy. Ichinose et al. (2021) found that muscle blood flow in the forearm microvasculature is significantly increased within 3 s post-contraction. However, rapid hyperemia and vasodilatory responses differ between the conduit artery and microvasculature (Ichinose et al., 2021). The magnitude of hyperemia is greater in the conduit artery compared to the microvasculature, and the time to peak hyperemia was shorter in the conduit artery compared to the microvasculature (Ichinose et al., 2021). Previous investigations have shown that NO plays a minimal role in matching oxygen delivery and oxygen demand during dynamic contractions (Rotarius et al., 2021), and rather oxygen tension (Ferreira et al., 2005) or mechanical deformation of erythrocytes (Shepherd et al., 2016) is responsible for FBF within the microvasculature. This quick reconciliation of the perceived overperfusion caused by the augmented post-contraction hyperemia may reflect significant reflex control of FBF within the microvasculature.

Tschakovsky et al. (1996) showed that a single muscle contraction has a higher blood flow when compared to single rapid cuff inflations mimicking a purely mechanical component. This would suggest that the muscle pump is not responsible for the total increase in blood flow but rather implies that both rapid vasodilation and the muscle pump work in concert to achieve the normal blood flow response. Our results agree with this assessment. The results of the present study indicate that FBF between conditions is not different prior to contraction following GTN administration. Upon contraction, FBF is significantly increased in retrograde fashion with GTN compared to control; and upon immediate release of the contraction, FBF is significantly higher with GTN compared to control. In addition, there were no changes to HR, SV, or MAP that might indicate a change in perfusion pressure. Positional differences, and therefore perfusion pressure, have been shown to play a significant role in the amount of reactive hyperemia following a single muscle contraction (Jasperse et al., 2015). However, there is no evidence in the present investigation that the perfusion pressure to the active muscle was different between CON and GTN. Thus, it appears that prior vasodilation of the feed artery has the potential to augment the skeletal muscle pump during mechanical contraction, as evidenced by significant increase in retrograde velocity. Upon release of contraction, rapid vasodilatory mechanisms may be exposed to greater shearing forces are augmented and result in a greater FBF. Tschakovsky and Sheriff (2004) proposed that the muscle pump may work by “charging up” arterioles by exposing them to higher-than-normal pressures during contraction. Upon release of contraction, the discharge of pressure would provide a larger than normal transient pressure driving flow across the arterial beds and into the veins (Tschakovsky and Sheriff, 2004). This effect of the muscle pump would be particularly brief. The findings of the present investigation provide support for this proposed mechanism. Specifically, the observed increase in retrograde velocity following GTN administration likely reflects an enhanced skeletal muscle pump function. The skeletal muscle pump functions to compress veins and venules during contraction and force blood from those segments. Upon release of contraction, the pressure gradient for blood flow is augmented (Laughlin, 1987). The resultant increase in shear stress may stimulate endothelial-mediated vasodilation. It is possible, in the present investigation, that the augmented retrograde flow during contraction reflects an augmented skeletal muscle pump, after which, the immediate post-contraction increases in FBF and FVC suggest a concurrent amplification of rapid onset vasodilation. Notably, this augmentation resolves swiftly (within 2 seconds of contraction release), consistent with the rapid characteristic of the muscle pump mechanism.

It is also possible that the significant increase in retrograde flow upon muscle contraction is due to the GTN itself. Previous research (Rotarius et al., 2021) has indicated that GTN causes a significant increase in retrograde blood velocity at rest and during muscle contractions, and a decrease in antegrade velocity during exercise. This led to there being no difference in FBF following rhythmic, dynamic exercise (Rotarius et al., 2021). Thus, within 10 s of dynamic exercise, FBF during GTN administration was equal to control contractions leading to the current investigation. It is possible that a withdrawal of parasympathetic nerve activity coupled with mechanoreceptor activation following a single muscle contraction contributed to the immediate resolution of the increased blood flow with GTN. Heart rate was increased in both conditions from 0 s to 2 s, which would suggest a withdrawal of vagal tone (Fagreus and Linnarsson, 1976). Additionally, the group III afferents in skeletal muscle have been shown to promote cardio acceleration and therefore could also contribute to the increase in HR seen following a single contraction (Nobrega and Araujo, 1993).

This study is not without limitations. First, the exercise intensity selected for the single contractions was 10% MVIC. This intensity was selected as it has been previously shown to be a high enough intensity to elicit changes in muscle blood velocity (Walløe and Wesche, 1988) without impacting central hemodynamic variables (Corcondilas et al., 1964). It is possible that higher contraction intensities would result in greater retrograde velocities, therefore potentially impacting the viability of the skeletal muscle pump, as evidenced by Walløe and Wesche (1988). Additionally, blood samples were not taken to assess endogenous ATP levels during contraction. As explained by Dillon et al. (2020), it is possible that regardless of the elevated blood flow prior to contraction, ATP may have been released from the mechanical deformation of erythrocytes. This may have contributed to the greater FVC following GTN administration compared to CON. Finally, it should be acknowledged that the mechanistic interpretations presented in this study are inferred from conduit artery blood flow and vascular conductance measures, without direct assessment of microvascular function, intramuscular pressure, or muscle compression forces. Therefore, while the present findings support a combined role of the skeletal muscle pump and rapid vasodilation, future investigations employing direct measures of microvascular responses and mechanical forces are required to more definitively delineate the underlying mechanisms.

## Conclusion

While many other studies (Brock et al., 1998; Corcondilas et al., 1964; Crecelius et al., 2013; Dillon et al., 2020) have employed the single contraction model, this study was the first to utilize GTN and the artery priming method to isolate the skeletal muscle pump. Each of these studies found that a “contraction-induced vasodilation” was responsible for the blood flow response to a single contraction. Our findings provide evidence to suggest that although the skeletal muscle pump may still be intact, and in fact augmented, as evidenced by the significant increase in retrograde flow during contraction, the increase in FBF and FVC immediately post-contraction indicates an augmentation of rapid onset vasodilation. These findings agree with more recent research exploring the effect of prior vasodilation on FBF using the single contraction model (Dillon et al., 2020; Perrin et al., 2026). It remains possible that the NO-mediated vasodilation prior to contraction may work synergistically with other putative vasodilators (K^+^, ATP, adenosine) to explain the augmented FBF response to a single contraction. It is important to note that the augmented increase in FBF post-contraction observed with GTN may not be attributed solely to enhanced rapid vasodilation or skeletal muscle pump. Rather, it reflects the combined influence of GTN’s effect on baseline diameter, a potentially greater pressure gradient following relaxation, and reduced downstream vascular resistance, all of which could contribute to the transient hyperemic response.

## Data Availability

The raw data supporting the conclusions of this article will be made available by the authors, without undue reservation.
